# Transformation of BTEX compounds emitted by aircraft engines at ground level

**DOI:** 10.1007/s11356-025-37247-w

**Published:** 2025-12-02

**Authors:** Jesús Rodríguez-Maroto, Rosa Pérez-Pastor, Susana García-Alonso, Enrique Rojas, David Sanz, Imara Ibarra, Manuel Pujadas, Dévora Hormigo, Jesús Sánchez, Paola Moreno, María Sánchez, Mark Johnson

**Affiliations:** 1 Centro de Investigaciones Energéticas Medioambientales y Tecnológicas (CIEMAT), 28040 Madrid, Spain; 2 Instituto Nacional de Técnica Aeroespacial (INTA), Torrejón de Ardoz, 28850 Spain; 3 Measurement and Engineering, Rolls-Royce Plc, Derby, UK

**Keywords:** VOC, BTEX, Ultrafine particles, Air quality, Aircraft, Airport

## Abstract

Volatile organic compounds, such as benzene, toluene, ethylbenzene and xylenes (BTEX) are emitted during the various aviation activities at ground level, such as take-off, approach and taxiing, that take place at or near airports. In addition to causing adverse health effects, these compounds are precursors of secondary aerosols (SOAs). The expected growth in air traffic in the near future makes it necessary to anticipate and control these emissions. As part of the AVIATOR project (EU Horizon2020), gaseous samples were collected in sorbent tubes and analysed by GC/MS, including emissions of commercial aircraft engines and airport ambient air, to study the evolution and transformation of BTEX. Three sites were selected: the INTA aircraft engine test cell, Ciudad Real and Madrid-Barajas airports. PM was collected on filters and substrates and analysed gravimetrically, with three different samplers: high-volume air sampler, Berner low pressure impactor, and an automated off-line sampler developed by CIEMAT. The ground idle configuration that simulates taxiing manoeuvres (before take-off and after landing) has been identified as a critical contributor to BTEX emissions at airports, with a concentration of over 460 ng L^−1^. Benzene is consistently emitted at higher levels than toluene and the emission of both increases with engine acceleration. In the plume, dilution with air decreases not only the concentration of BTEX, but also the prevalence of the compounds, making benzene no longer the dominant compound. Variations in diagnostic ratios and meteorological conditions, as well as sensitivity parameters to PM concentration, may suggest physicochemical transformation of BTEX into SOAs.

**Supplementary Information:**

The online version contains supplementary material available at 10.1007/s11356-025-37247-w.

## Introduction

The acronym BTEX corresponds to a group of aromatic volatile organic compounds (VOCs) including benzene, toluene, ethylbenzene and xylenes (o-, m-, and p-isomers). The adverse health effects of benzene are well documented and it is classified as a known human carcinogen (Group 1) by the International Agency for Research on Cancer (2004). The European Union (E.U.) has established an annual limit for benzene in ambient air of 5 µg m^−3^ (European Parliament, 2000).

BTEX compounds are important precursors of secondary organic aerosols (SOAs) through photochemical oxidation reactions in air (Johnson et al. 2005, Martín-Reviejo and Wirtz 2005, Ng et al. 2007, Henze et al. 2008). They are found in fossil fuels and are primarily emitted in the combustion process, being the industrial, domestic, and transport sectors their main anthropogenic sources. Other minor sources include gasoline evaporation, solvents, and paints (Masiol and Harrison 2014). In the transport sector, aviation emissions also contribute to ground-level air pollution by BTEX.

Aviation emissions impact the atmosphere both at ground level around airports and at cruising altitudes affecting the upper troposphere and lower stratosphere. According to the International Civil Aviation Organisation (ICAO), landing and take-off (LTO) cycles include all phases of flight below 3,000 feet above ground level (AGL), such as take-off, approach and taxiing. The Federal Aviation Administration (FAA) forecast that air traffic will increase by 2030, highlighting the need to assess the influence of aviation emissions on air quality with particular attention to organic compounds (FAA, 2009).

Various studies have examined VOCs emissions related to aircraft operations at airports. Aromatic compounds in jet fuels can be emitted as unburned material, by products of incomplete combustion, or from fuel evaporation and refuelling processes (Spicer et al. 1992; Schürmann et al. 2007; Levy et al. 2008; Knighton et al. 2009). VOCs have been measured in various scenarios: such as test cells, airports (from aircraft engines or exhaust plumes) and in-flight measurements, although there is still a significant gap in understanding the relationship between emitted compounds and their final fate within the airport environment.

VOCs emissions vary according to engine operation conditions, generally decreasing with power increases due to more efficient combustion, despite increased fuel consumption. However, some evidences suggests that aromatic compounds and alkenes may be emitted in greater quantities at higher engine power (Anderson et al. 2006), similar to soot emissions (Warnatz et al. 2006). The limited studies conducted in these complex scenarios, often constrained by strict safety standards, contribute to data discrepancies and uncertainties.

Aircraft exhaust is just one of several emission sources at an airport. Ground support equipment and related vehicles also contribute to emissions (Bendtsen et al. 2021). Additionally, power generation plants, industries, roads and nearby population centres must be considered.

A review on the contributions of airports to ambient pollution concluded that there is insufficient knowledge about the health impacts of VOCs related to these emissions (Masiol and Harrison 2014).

Several methods have been used to identify VOC sources in ambient air. Different values of diagnostic ratios, such as benzene to toluene (B/T) and m + p xylenes to ethylbenzene (X/E), are indicative of nearby traffic emission, photochemical ageing, and long range transport (Miller et al. 2011). The B/T ratio in air remains relatively constant close to emission sources, and. can change over time and with the atmospheric processing of air masses. The X/E ratio can serve as an indicator reflecting the extent of photochemical reactions, so that higher and lower X/E ratios would suggest fresh VOC mixture coming from near sources and polluted air advected from external areas, respectively (Liu et al. 2024).

Given the role of the photochemical oxidation of BTEX in SOA formation, it is crucial to investigate these relationships, however, the contribution of these compounds to SOA is still far away from being understood, as there are involved very complex processes.

Yang et al. (Yang et al. 2020) found that the increase in VOCs emission does not result in increasing SOA generation in a linear manner. They also reported that the tenfold increase in VOCs emission seems to increase 30% in SOA formation.

Wei et al. (Wei et al. 2022) found that in the conditions of stronger solar radiations (summer) and low-middle aerosol loads, VOCs chemistry plays an important role in PM_2.5_, increasing through contributing SOA; but in the conditions of lower solar radiations (winter) and heavy aerosol loads, VOCs chemistry was greatly weakened and had the minor impact on PM_2.5_ increasing.

A study on BTEX emissions from aircraft engines and immission at an airport was carried out. Previously, a study on organic compounds in particulate matter was published (Rodríguez-Maroto et al. 2024), both included in the AVIATOR project. BTEX measurements were taken at ground level simulating LTO cycles, at three locations: the aircraft engine test cell (engine exit plane and stack) at the Instituto Nacional de Técnica Aeroespacial (INTA), the engine exit plane of an AIRBUS 340–600 at Ciudad Real International Airport (CRIA), and the ambient air at Madrid Barajas International Airport. The goal was to characterise emissions under different engine and fuel conditions, relate them to air quality measurements at an airport and find their relationships with particle matter (PM), providing insights to inform future pollution control policies.

## Experimental

### Sampling locations

Three locations were selected for studying BTEX from aviation activities in ambient air at ground level (AGL): INTA aircraft engine test cell, Ciudad Real and Madrid-Barajas airports.

At INTA´s facility, A Rolls-Royce Trent 500 engine was used for the test. Two sampling points were selected, the engine plane and the exhaust stack of the test cell. In addition, at CRIA, a dedicated sampling probe was positioned in the engine exhaust plane of one of the four engines of an AIRBUS 340. In both locations, the aircraft engine was tested under various operational configurations: ground idle, ground idle to take-off and flight idle, adapted to the different operations of an aircraft at the airport. Finally, at Madrid-Barajas, ambient air was measured between two runways.

The first configuration, ground idle, refers to the airplane being stationary with the engines running or taxiing at low speed on the runway. During take-off stage, the aircraft reaches maximum acceleration, approaching 100% of the nominal thrust. After take-off, the ascent operation begins, during which engine power is reduced. Flight idle refers to approach phase before landing.

In each of the selected turbofan engine configurations for the aircraft, certain key control parameters needed to be adjusted during testing. These included the speeds of the compressor and low-pressure turbine (%N1), the high-pressure compressor and turbine (%N2), and the fan (%N3). Additionally, factors such as temperature, pressure, and the position of the exhaust valve were also varied. These adjustments affected changes in emission characteristics, even when the fuel consumption for a specific configuration remained constant. CO₂ concentration was used as a reference parameter, since in combustion processes it varies depending on the engine configuration. As a result, variations in the primary and secondary flow could still impact emissions.

The emission at the engine exit plane correspond to the primary "core" flow. Instead, in the test cell stack there are three contributions to the exhaust flow: the primary flow, the secondary flow (caused by the compression of air entering the engine through the fan in the downstream compressor) and the tertiary or entrained air around the engine, which is incorporated in the exhaust plume. The fourth and last flow that is incorporated into the exhaust is excess breather oil.

Ambient air samples were collected at Madrid-Barajas airport, located 12 km northeast of the city of Madrid. The sampling site was located between the two northern runways of the airport, approximately 700 m from runway 18R/36L and 600 m from runway 18L/36R. These runways are oriented in the north–south axis and were used for departures almost all days during the campaign. Terminal buildings T4S and T4 were located to the south and southwest of the sampling site (Infopower_Plant_Report 2006).

### Sampling procedures

Sampling procedures were similar at the three selected sites. A gas emission sample was taken from the aircraft engine exhaust (engine plane at INTA and CRIA and stack at INTA) and the airport ambient air, using the appropriate sampling probes and passed through the transfer lines to the Off-line Automatic Sampler (OAS), designed and manufactured at CIEMAT for the AVIATOR project (H2020) (Rodríguez-Maroto et al. 2024). The samples were collected on sorbent tubes for further analysis. Sampling rate and time were variable according to the different scenarios and experiments, achieving gas volumes from 4 to 18 L. Sampling time at CRIA was between 20 and 30 min at a flow rate of 0.2–0.5 L min^−1^; time of sampling during INTA experiments was between 15 and 30 min, flow rate of 0.4–0.6 L min^−1^Ambient air at Barajas Airport was taken during 30 min at a flow rate of 0.6 L min^−1^.

At the first location, the INTA Turbojet Test Cell, an “Emission Traverse Probe" (ETP) was used for sampling in the engine plane (Aragón et al., 2018) and a multi-hole probe located in the exhaust stack of the facility, both designed and manufactured at CIEMAT (Rodriguez-Maroto et al. 2016; Rojas-García et al. 2019). A conventional probe from Manchester Metropolitan University and Scitek (Derby UK) was used at Ciudad Real International Airport, CRIA, which consists of two parallel tubes, one to sample gases and the other to sample particles. A conventional single-tube probe was used for environmental measurements at Barajas airport. The transfer lines length is variable depending on the location.

To understand the obtained data, ambient data such as temperature, humidity and wind speed were also recorded (see Tables 1S and 2S, ESM).The OAS is an automatic sampling system, with the ability to operate in remote mode. It allows the simultaneous or sequential collection of multiple gaseous and particulate samples for subsequent laboratory analysis (post-sampling analysis). Emission samples were initially diluted with filtered, dry air to lower their temperature below 100 °C. Additionally, the OAS was fitted with a temperature controller in the area where the sampling tubes were located, conditioning the samples to temperatures between 20 and 30 °C. This prevented reactions that might occur at high temperatures and the condensation phenomena associated with low temperatures. Samples to analyse BTEX were taken into commercial sorbent tubes supplied by Markes International Ltd (UK) filled with Tenax TA/Carbograph 1TD/Carboxen 1003 (C3-AAXX-5266). A second tube in line was set during the engine test cell experiments (INTA and CRIA) to evaluate the breakthrough*.* After sampling, the tubes were capped and stored at 4 °C until analysis in the laboratory.

### BTEX analysis

Samples were subsequently analysed using a gas chromatograph/mass spectrometer, GC/MS (Agilent 6890/5975B, Santa Clara, USA) coupled to a thermal desorption, TD (Unity, Markes).

Ambient air, stack and breather samples were thermally desorbed using helium as purge gas. Desorption parameters: pre-purge 6 min at a flow rate of 50 mL min^−1^; primary desorption at 280 °C for 5 min at a flow rate of 30 mL min^−1^, no inlet split; cold trap low 25 °C; pretrap fire purge 3 min at 50 mL min^−1^; cold trap high at 300 °C for 6 min; outlet split 10 mL min^−1^ and flow path temperature 200 °C. The Air Toxic Analyser cold trap (Markes) was used.

Engine samples were desorbed in the same way, but applying an outlet split of 200 mL min^−1^ in order to avoid saturation of the detector.

Separation was achieved using a DB-624-MS column (60 m × 0.25 mm ID × 1.40 µm film thickness) (J&W Scientific, USA). The column oven temperature was maintained at 40 °C for the initial 5 min, then it was increased at 6 °C min^−1^ up to 220 °C and held for 3 min. The injector port was set at 250 °C and the transfer line from TD to GC at 300 °C. Samples were injected in splitless mode, using helium as carrier gas with a flow rate of 1.0 mL min^−1^. MS was operated in “Electron Ionization” EI mode at 70 eV. The ion source temperature was 230 °C and the quadrupole temperature 100 °C, operating in the SCAN mode (50–250 m/z).

Calibration was achieved using liquid standards in methanol, 5 µL of which were injected in clean sorbent tubes through a calibration loading rig (Markes) while purging with nitrogen for 10 min.

A commercial standard mixture of BTEX 100 μg mL^−1^ in methanol (Dr. Ehrenstorfer GMBH) was then diluted to inject solutions in the range 0.5–150 ng per tube for each compound.

Field blanks were taken (n = 24) during the whole monitoring periods, and data were blank corrected.

The method detection limits (LOD) for each compound were calculated as three times the standard deviation of seven spiked tubes (0.5 ng), being the obtained values below the first calibration level. LOD were in the range 0.2–0.4 ng.

Precision, determined from five replicates analysis of a standard of 2.0 ng was within 7% for o-xylene to 16% for m + p xylenes. Tubes were submitted to a second desorption in order to check recovery. Even in the most concentrated samples, a recovery efficiency > 99% was achieved for all compounds.

### Sources identification of BTEX

The change of mixing ratios of different species of VOCs are affected by both photochemical processes and emission inputs, so these ratios are used to provide preliminary information on their emission and transport.

Specifically, the ratio benzene-to-toluene B/T has been extensively used to source identification as they are present in different proportions according to emission source (Schürmann et al. 2007; Cui et al. 2022).

Benzene and toluene are highly correlated with vehicular emissions; however, toluene has other sources such as solvent evaporation, whereas benzene is not commonly present in solvents. Nevertheless, the lifetime of toluene in the atmosphere is five times shorter than that of benzene, due to the higher reactivity with the OH radical, so it depletes more quickly than benzene, resulting in variations in the ratios (Jung et al. 2011).

In areas heavily impacted by vehicle emissions, this ratio lies in the range of 0.45–1.1. According to traffic emission studies, a typical value for automotive exhausts is around 0.37. Ratios less than 0.11 have been reported for solvent use and industrial processes (0.17–0.7). In burning source emission studies, a ratio above 1.7 was deduced in different processes (Cui et al. 2022). So, generally, a ratio around 0.5 indicates a strong influence of vehicular emissions in BTEX concentrations, while lower values may indicate that BTEX are associated with solvent evaporation related to other sources such as industrial facilities and regional sources (Wang et al. 2014; Cerón-Bretón et al. 2018).

To examine aging degree of air masses, the reactivity differences among typical VOC species that have similar sources but different chemical reactivity are used. The atmospheric lifetimes of benzene and toluene are 12.5 and 2.0 days, respectively, which are rather stable, while those of m,p-xylenes, and ethylbenzene are 3 and 8 h, respectively (Liu et al. 2008). Thus, the ratio X/E can be used as an indicator of the photochemical aging of air masses because of their similar sources in urban environments and differences in atmospheric lifetimes, implying X/E ratio would decrease during transportation from the source (Cui et al. 2022). A value around 3.6 has been reported as the typical emission ratio of these compounds (Nelson and Quigley 1983). Ratios measured in urban atmospheres are around 3 (Yurdakul et al. 2018). High values of these ratios (those closer to their expected emission ratio) typically indicate fresh local emissions, whereas low values suggest that the site is being influenced by emissions originated some distance away, aging of the air mass and effects of photochemical reaction. Ratios substantially less than 3 indicate the transport of VOCs from distant sources. So, a lower X/E ratio indicates the aging of VOCs in the atmosphere and it can diagnose the effects of local pollution, transport, or photochemical reactions (Spicer et al. 1984; Presto et al. 2011; Jathar et al. 2012; Kim et al. 2019).

In terms of aircraft emissions, very few data are available. Spicer et al. (Spicer et al. 1984) established that a B/T ratio of 1.6 is typical in aviation kerosene exhaust during taxiing. On the other hand, Presto et al. (Presto et al. 2011) through emission factor calculations, reported that the B/T ratio varies between 2.7 and 3.1 at loads of 4 and 7%, while the X/E ratio was of 6.7. Jathar et al. (Jathar et al. 2012) analysed different fuels and found that the B/T ratio ranged from 2.5 to 3.6 during idle, taxi, landing and take-off stages, while the X/E ratio showed considerable variability, with values between 1.4 and 8.0.

### Relationship between BTEX and Particulate Matter PM

The role of volatile organic compounds (VOCs), particularly aromatic hydrocarbons, as precursors in secondary organic aerosol SOA formation is well established, as mentioned above. Regarding relationship between BTEX and PM, Han et al., (Han et al. 2018) first established the concept of VOC-sensitiveness (VOC-S) coefficient to investigate the quantitative relationship between different groups of VOCs and PM_2.5_ concentrations.

1 $$VOC-S=\frac{\Delta VOCs/BVOCs}{\Delta P{M}_{2.5}/B{PM}_{2.5}}$$

The authors defined a PM_2.5_ background (BPM_2.5_) of < 5 µg m^−3^, establishing successive ranges in 5 −10 µg m^−3^ intervals, being the background of VOCs, BVOCs, the measured VOCs corresponding to these BPM_2.5._ ΔVOCs and ΔPM_2.5_ correspond to the difference between the measured concentrations of VOCs and PM_2.5_ and those of BVOCs and BPM_2.5_, respectively. This same concept was also applied to evaluate the influence of different families of VOC on PM_2.5_ in a representative industrial city of Korea (Lee et al. 2023).

A VOC-S significantly greater than 1 indicates that a large increase of ΔVOCs is required to change the unit of PM_2.5_. Conversely, coefficients lower than 1 imply that PM is easily affected by small changes in ΔVOC concentrations. If the measured VOC concentration is less than the BVOC (background VOC level), it is not possible to compute the VOC-S.

In our study, the same parameter defined by Hans but adapted to the BTEX has been considered:

2 $$BTEX-S=\frac{\Delta BTEX/BBTEX}{\Delta PM/BPM}$$

where ΔBTEX and ΔPM represents the difference between the measured concentrations of BTEXs and PM_2.5_ and those of BBTEXs and BPM_2.5_.in a specific PM range, respectively.

In this equation, BPM refers to the lowest measured PM concentration (always greater than 5 µg/m^3^), and BBTEX is the corresponding BTEX concentration at that PM level. This approach enables us to quantify the sensitivity of PM to BTEX fluctuations and assess the impact of BTEX emissions on particulate matter concentrations in the ambient air. By understanding this relationship, we can gain insights into the contribution of BTEX to PM formation, which is essential for developing more targeted air quality control strategies.

## Results

Results are presented as follows:

- 3.1 Measurements at ground level, AGL (direct emissions and inmissions near runways).
- 3.2 BTEX ratios for diagnosis.
- 3.3 Relationship between BTEX and PM

### Measurements AGL

Measurements of the engine exit plane (INTA and CRIA), the test cell stack (INTA) and the airport runway environment (Barajas) are included.

#### Engine exit plane

- Engine Test Cell (INTA)

The study conducted in the Engine Test Cell simulated five typical configurations of the operation of airplane engines at or near an airport, according to ICAO:

- Ground Idle, GI during taxiing (pre-take-off and post-landing),
- Ground Idle to Take-Off, GI to TO (previous to take-off, the engine is gradually accelerated),
- Take-Off, TO (at full throttle)
- Take-off to Climb-Out, TO to CO (ascent to reach 3,000 m)
- Flight Idle, FI (approach from 3,000 m or landing).

**Fig. 1:**
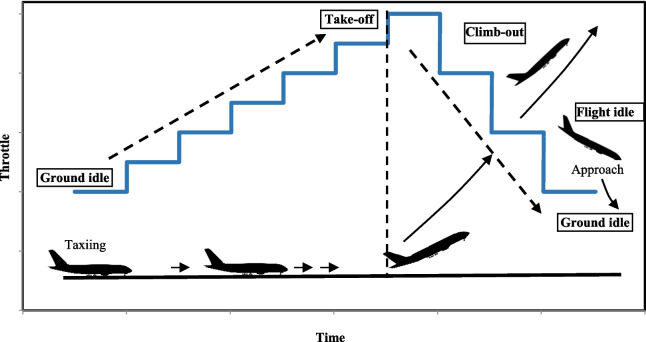
Engine power step curve, simulating different operations of airplane

Figure 1 shows a simple diagram to illustrate these transitions. GI to TO was simulated using a stepped acceleration curve, while the transition from TO to CO and FI was simulated using deceleration steps.

The results of the four assays implemented in the Test Cell are presented in Table 3S (ESM). BTEX measurements were taken during a test simulating take-off using an acceleration curve (from GI up to TO) and using a deceleration curve (from TO to CO).

These results reveal substantial variations in BTEX emissions for the same operating configuration. These differences are due to variations in operating and control parameters, such as high and low-pressure, set by the engine manufacturer for each test. The different operating configurations that were repeatedly implemented affect combustion and secondary airflow dilution. Additionally, measurements taken on different days, under varying ambient conditions, and with different engine configurations within the same type of test contribute to the non-uniformity of the data (Fig. S1, ESM).

Despite these uncertainties, the benzene concentrations consistently exceeded those of other compounds in all test configurations, followed by toluene. Benzene and toluene concentrations were nearly two orders of magnitude higher than that of ethylbenzene, in some cases, which was typically the lowest among the six compounds measured. This occurs in operating modes that simulate, on the one hand, the transition from TO to CO and, on the other hand, that of TO. (Spicer et al. 1984).

BTEX emissions were higher in the GI mode compared to the TO mode, with intermediate operating modes showing intermediate BTEX emissions. This pattern suggests that lower engine loads (e.g., ground idle) produce higher BTEX emissions as direct consequence of an incomplete combustion and reduced secondary airflow dilution.

(b) AIRBUS 340–600 Engine (CRIA)

At CRIA, BTEX emission from one of the engines of an AIRBUS 340 was measured in two different campaigns (winter and summer) and for two types of fuel: the standard commercial aviation fuel (Jet A1) and a blend of Jet A1 and Sustainable aviation fuel of synthetic origin (Jet A1 + SAF) (Table 2S). During the summer, two operating configurations were tested using Jet A1: flight Idle and ground idle (65% N3). During the winter, in addition to the test of the flight idle configuration with Jet A1, the following configurations were tested with the Jet A1 + SAF: flight idle, 30% N1 and ground idle (65% N3). In three-shaft turbojets, N1 refers to the rotation speed of the low-pressure compressor expressed as a percentage of the maximum design, N2 to that of the medium pressure compressor and N3 to that of the high-pressure compressor. These parameters were monitored in the airplane cabin.

For both fuels (Jet A1 and Jet A1 + SAF), the GI configuration (62–65% N3) produced the highest BTEX emissions either in winter and summer, being benzene and toluene particularly significant (Table 4S, ESM). Consistent with the test cell results at INTA, the configuration with the lowest BTEX emissions was 30%N1 (which corresponds to a low-thrust setting used during engine start up to verify smooth operation). At CRIA, it was not possible to measure BTEX emissions during take-off.

The BTEX emission in the FI configurations in winter was found to be slightly lower than in summer with JET A1. The BTEX produced with the JET A1 + SAF mixture is slightly higher than that of JET A1 for FI. The lowest BTEX values were measured in the start-up configuration (Fig. S2, ESM).

**Fig. 2:**
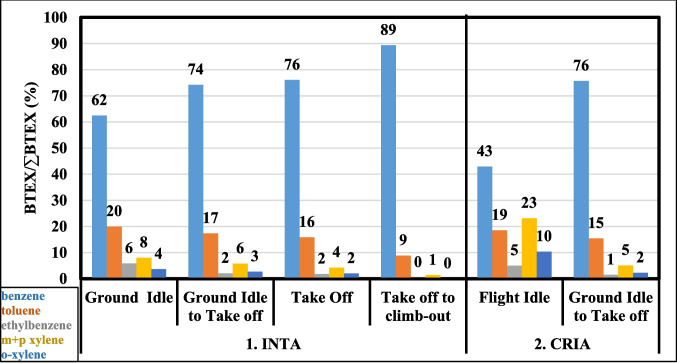
BTEX/∑BTEX ratio different operating configurations at INTA and at CRIA

To assess the impact of different operating configurations on each BTEX compound, we calculated the ratio of each compound to the total BTEX concentration (BTEX/∑BTEX) for each test configuration. Figure 2 displays this ratio, which was derived from the average concentrations measured in the engine exit plane for each operating configuration tested using Jet A1, both in the INTA test cell and in the AIRBUS 340 engine at CRIA.

This approach provides information on how specific conditions (engine operating mode, fuel type, and even environmental factors) can influence the emissions profile. This metric is essential for determining which configurations can lead to higher proportions of certain BTEX compounds, helping to identify possible sources and patterns in BTEX emissions.

The percentage of benzene increased from the idle setting on the ground to the climb phase, while the percentages of the other BTEX compounds decreased in the configuration tested at INTA. This suggests that higher engine temperatures favour the formation of lower-molecular-weight compounds, resulting in higher benzene concentrations and lower concentrations of the other compounds.

When comparing the BTEX/ΣBTEX ratios for a same configuration between the two scenarios, INTA and CRIA, the results were similar (e.g. ground idle to take-off). Consequently, it can be confirmed that the operating configurations, ordered from lowest to highest benzene emission, are as follows: flight idle < ground idle < ground idle to take-off < take-off < take-off to climb-out. The remaining BTEX compounds were generally emitted in reverse order. Notably, the m + p xylene ratio was higher in the flight idle configuration.

#### Stack emission test cell (INTA)

The stack emission consisted of the primary and secondary flows of the engine, along with a tertiary flow formed by the air entrained around the engine. Occasionally, there was a fourth flow due to excess oil from the breather. Consequently, the primary flux measured at the engine exhaust plane was diluted by ambient air from the secondary and tertiary fluxes in the stack emission. A dilution factor 6–7 could be estimated from the aerosol particulate measurements performed between the engine plane (approx. 1.1 mg.m^−3^) and the stack (approx. 0.17 mg.m^−3^). Additionally, the breather flow could contribute excess oil to the stack emission.

Three engine operating configurations were tested during four days assays: GI, GI to TO, and TO (Table 5S, ESM). From the results, it was revealed that the BTEX concentrations measured in the stack did not follow a clear order of prevalence in the engine plane emissions. More specifically, the order of BTEX concentrations in the measured engine plane emissions (benzene > toluene > m + p xylene > ethyl benzene > o-xylene) was not consistent in the stack emissions. In this case, while benzene, toluene, and m + p xylene remained dominant, their order varied randomly for a same configuration.

The PM dilution pattern that coincides for all three configurations was not observed for BTEX, suggesting that these compounds, from their origin to the stack measurement, have already undergone some physicochemical transformation, in which PM could be involved (Fig. S3, ESM).

The lower temperature of the ambient air (20–25ºC) compared to the emissions (< 500ºC) could contributed to transformations of the emitted BTEX.

Minimal differences were observed in the concentrations in the stack emission when the engine configuration is changed. Additionally, sometimes the concentrations of the higher molecular weight compounds are similar to those of the more volatile ones (Fig. S4, ESM).

The dilution of primary emission by entrained air in the stack exhaust would typically result in a proportional decrease in the concentration of each BTEX compound in the measurement. During the tests there was no risk of smoke re-ingestion in the test cell because the wind in the area left the entrance of the installation upwind of the stack.

However, this was not consistently observed, suggesting that some compounds may undergo physical or chemical reactions altering their original concentrations. This phenomenon is expected to occur similarly in aircraft engine exhaust plumes during landing and take-off operations.

In stack emissions, xylenes (m + p and o-xilenes) were notably present, comprising in some cases over 40% of the total BTEX concentration, being more than four times higher than that measured in the primary emission.

In the TO configuration, the order of prevalence differs from the primary emissions, because benzene is no longer the major BTEX compound, unlike in the other configurations where the order of prevalence is maintained.

It was observed that the concentrations of each BTEX compound from the primary emission to stack not only decrease (dilution factor ≈10) due to dilution by the secondary and tertiary air flows, but also evolve differently. Table 1 shows the ratio between the concentrations of each compound measured at the two measurement points, engine level and stack at different engine operating conditions.

**Table 1:** Ratio of BTEX compounds measured at the engine plane and the stack

Operating condition	BTEX engine/BTEXstack				
	Benzene	Toluene	Ethylbenzene	m + p xyilene	o- xylene
GI	125	52	85	24	20
GI to TO	20	7	3	3	2
TO	56	8	2	2	1

As can be seen, highest BTEX levels were measured in the ground idle configuration. In more detail, while benzene decreased more than 100 times from one measurement point to the other, the xylene decreased only 20 times. In the case of take-off configuration, the ratio was more than 50 times higher for benzene than for o-xylene. Therefore, for each configuration, the ratio between the concentration of a BTEX compound in the primary emission and that in the stack emission decreases with the molecular mass of the compound or its lower volatility.

This suggests that the more volatile compounds could be adsorbed onto primary particles in a relatively fast process, or be oxidized by active radicals at a high temperature. However the time elapsing from their emission into the stack would be very short (just over one second) (Tully et al. 1981; Schöbel et al. 2001; Zhang et al. 2019). Already in the environment, they would undergo the processes of formation of known secondary organic aerosols (SOA). This is consistent with the findings of Kilic et al. (Kılıç et al. 2018), who determined that over 90% of the SOA mass was organic and could largely be attributed to the oxidation of BTEX and other organic gases.

Under atmospheric conditions, BTEX may undergo oxidation reactions with ozone (O_3_), nitrate radicals (NO_3_), and hydroxyl radicals (OH), leading to the formation of less volatile products. These products may further react and/or partition into the condensed phase, resulting in complex chemical composition profiles within the aerosol (Izumi and Fukuyama 1990, Srivastava et al. 2022; Iyer et al. 2023).

#### Environmental measurements around runways at Barajas airport

From October to December 2021, a total of eighty-five ambient air samples were collected around the runways of Barajas airport to determine BTEX. Sampling was conducted on eighteen days in October and nineteen days in November and December. Three samples were collected daily on each sampling day: at 12:00 (morning), 20:00 (evening), and 4:00 (night) (Fig. S5, ESM).

The data obtained at Barajas Airport, both in the warmer and colder months, show BTEX concentrations in the same magnitude order to those obtained at the INTA stack during the tests, which were carried out in summer with the different LTO cycles. In the cooler months, BTEX appeared to be in lower concentrations in the airport environment, as expected.

The off-line sampler (OAS) facilitated simultaneous collection of gases and Total Suspended Particulate Matter (TSP), while PM_10_ and PM_18_ samples were collected using the High-Volume Sampler (HVAS), and the Berner Low-Pressure Impactor (BLPI), respectively (Tables 6S and 7S, ESM).

The meteorological parameters and flight activity appear to influence the BTEX and PM concentrations. Under southerly wind conditions, aircraft emissions at Barajas airport were frequently transported northward instead of toward the measurement points, reducing their collection efficiency. Conversely, during calm periods with wind speeds below 1 m.s^−1^, emissions localized within to the airport area, leading to elevated pollution levels and increased concentration of both BTEX and PM in the ambient air (Fig. S6, ESM).

**Fig. 3:**
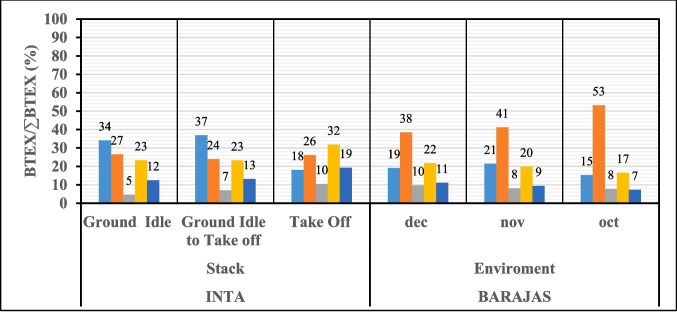
Comparison of BTEX/∑BTEX ratios in measurements at INTA and Barajas

Figure 3 compares the BTEX/∑BTEX ratios for the test cell stack (INTA) in each operational configuration tested, and those of Barajas airport. Calculations have been performed with mean values of the measured concentrations for each configuration at INTA and the monthly averages for each measurement period at the airport.

Although the ratios for GI and GI to TO configurations at INTA stack, align with those at plane engine in Fig. 2, they differ in their values. The take-off configuration notably deviates even in the order of prevalence. For instance, while the benzene/∑BTEX ratios for the ground idle and ground idle to take-off configurations were 62% and 74%, respectively, in the engine plane measurements these ratios were markedly lower in stack measurements (34% and 37%, respectively).

As in all measurements at INTA both at the engine plane (same in CRIA) and in the stack, with the exception of the take-off configuration measured in the stack, benzene and toluene showed the highest concentrations. In the take-off configuration, higher molecular weight compounds become more significant, with m + p xylene predominating over others. Furthermore, in this configuration, the (benzene/∑BTEX) and (ethylbenzene/∑BTEX) ratios observed in INTA stack measurements closely resemble those found at Barajas Airport, probably due to the proximity of the measurement point and the take-off area of the airport. Although the toluene/∑BTEX ratio at Barajas was higher, likely due to additional emission sources, Fig. 3.

**Fig. 4:**
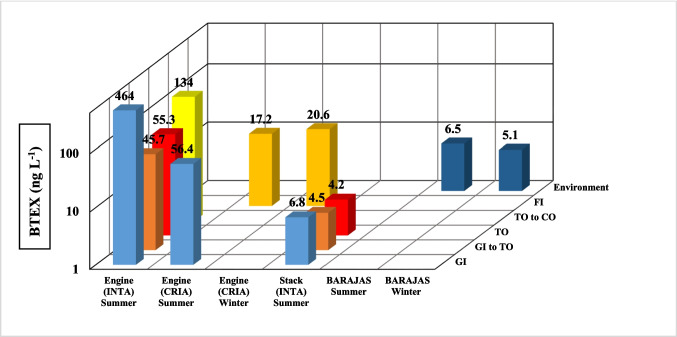
BTEX concentrations from engine and stack emissions (INTA and CRIA) and environment (Barajas)

Figure 4 presents the average concentrations of BTEX emissions for the various configurations, measured at the engine exit plane (INTA and CRIA) and test cell stack (INTA), as well as in the ambient environment at Barajas Airport.

The data reveal that BTEX emissions measured in the INTA in the idle configuration (GI) were approximately ten times higher than the configurations: acceleration, ground idle to take-off, (GI to TO) and take-off (TO). This is slightly higher than the GI to TO configuration. BTEX emission in GI exceeded almost three times that of flight idle (FI).

Engine plane measurements at CRIA for the two test configurations that could be measured, GI and FI, show lower BTEX concentrations compared to those determined at INTA for the GI configuration. Moreover, the concentrations at FI were lower than GI at both testing locations.

A pronounced decrease in BTEX concentration is observed from the engine plane to the stack: while the GI to TO and TO configurations showed a decrease of about a factor of 10, the GI configuration experienced a decrease by nearly one hundred. This suggests that the physical and chemical transformation of BTEX may start early in post-emission.

Ambient measurements at Barajas airport closely mirrored those of the stack emissions, indicating that the BTEX transformation could be initiated and largely carried out within the emission plume before its complete dispersion into the ambient air. These transformations may vary for each BTEX compound. Therefore, investigating the relationship between these compounds is crucial for understanding their behaviour in such environments.

### BTEX ratios

The benzene/toluene (B/T) and m + p xylene/ethylbenzene (X/E) ratios were calculated for the three types of measurements performed in this study: Plane engine exhaust emissions at INTA and CRIA (Table 8S, ESM)) and Stack emissions (INTA) and ambient air at Barajas airport (Table 9S, ESM)). The measurements at the engine exit plane, indicated that the concentration of benzene and xylene can be up to six times higher than those of toluene and ethylbenzene respectively. This would mean that the B/T and X/E ratios would be in the range of 1 to 6 in most cases, varying depending on the test configuration. The B/T ratios showed a minimum of 0.06 and a maximum of 10.07 (INTA), this last value corresponded to the simulation of the ascent operation (TO to CO). The X/E ratio has a minimum of 0.50 and a maximum value of 5.50, and only one of the configurations resulted in values lower than one for both the B/T and X/E ratios, due to the higher acceleration of the engine.

For the emissions of the engine tested at CRIA, the B/T ratios ranged from 2.05 to 5.33 and the X/E ratio between 1.00 and 5.13. Therefore, the values of the B/T and X/E ratios obtained from the engine plane measurements are mostly in the range of 1 to 6, similar to previous measurements (Presto et al. 2011; Jathar et al. 2012).

The values of the B/T ratio in the INTA stack were lower than those determined in the engine, ranging from 0.15 to 3.81, while those of X/E were much higher, reaching values greater than 20, with a minimum of 4.63. In short, the values of the B/T and X/E ratios would be in the intervals (0–4) and (5–20), respectively. Schürmann et al. (Schürmann et al. 2007) measured the exhaust plume at different stages (ignition, idle, taxing) in the airport of Zurich. A ratio well below 1 was found for refuelling emissions and engine ignitions, while it was about 1.7 for exhausts from airplane. Guimares et al. (Guimarães et al. 2010) sampled at the idle and taxi areas of the Santos Dumont National Airport, Rio de Janeiro. These authors reported a mean B/T ratio of 0.55 which probably reflects the composition of emissions during the engines ignition period, when the engines have not reached their final temperature as compared to those results obtained by *Schürmann* (Schürmann et al. 2007).

The general trend shows, the ratio B/T decreases, while X/E increases, significantly for some test conditions. As stated above, if there were only a dilution of BTEX in the path from the engine exhaust to the stack, the B/T and X/E ratios would be the same as at the engine plane. Therefore, the observed variations point to physicochemical transformations in the exhaust plume. A rapid benzene oxidation could occur in highly oxidizing regions of the plume, with a high presence of oxygenated radicals (-OH, -O, -HO₂) and high temperatures. In this way, the reactivity between toluene and benzene could be reversed (Tully et al. 1981; Schöbel et al. 2001; Zhang et al. 2019). This could explain the decrease in the B/T ratio from the exhaust emissions to the stack.

Also, the ratios determined in the measurements made at Barajas airport were between 0.04 and 4.14 for the B/T ratio and between 0.80 and 11.40 for the X/E ratio. The former were in the same range as those obtained in the stack, while the latter were lower. These ratios decreased from the warmest to the coldest periods, so that the range of variation of the B/T ratios in October was from 0.04 to 4.14, and in November and December they ranged from 0.13 to 1.75. However, the values of the X/E ratio in October are included in the range from 1.00 to 3.08 and in November and December, from 0.80 to 11.40. This suggests that additional sources at the airport may be providing toluene and ethylbenzene in higher proportions than benzene and xylene respectively, especially in the colder months.

**Fig. 5:**
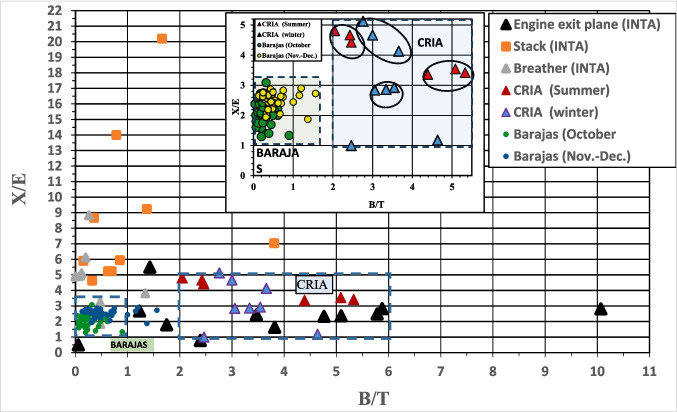
Benzene/toluene (B/T) and m + p xylene/ethylbenzene (X/E) ratios for all scenarios studied. In the zoom only the results of the measurements at CRIA and Barajas airports are included

To better understand the possible transformations of BTEX compounds, the Fig. 5 illustrates the B/T and X/E ratios for the different scenarios. Breather ratios have also been included for the INTA trials. The data corresponding to the motor plane for the same operating configurations have been included in the same ellipsoidal enclosure (black line).

Regarding the B/T ratio, it is easy to observe that the lower values corresponds to the measurements in the air at Barajas airport, and coincides with those of the INTA test cell stack. Conversely, the higher values are observed for the measurements in the engine plane at both INTA and CRIA. As for the X/E ratio, very high values corresponded to the INTA stack measurements, possibly to the contribution of breather, while the lower values corresponded to the environmental measurements at Barajas airport. Slightly higher values are those at the engine plane, depending on the test configuration, and at CRIA.

According to these relationships (ap 2.3), the decreasing trends would diagnose the effects of local pollution, transport or photochemical reactions. Such variations in the different study scenarios would confirm the transformation of BTEX compounds from their origin in the engine plane to the airport environment.

The B/T ratio increases progressively from GI to TO and CO operations (\* MERGEFORMAT Fig. 2), which is consistent with measurements made in the vertical profile around Beijing airport by Liu et al. (Liu et al. 2013). Although this vertical distribution has been attributed to diffusion and photochemical consumption of BTEX, the present study provides evidences to relate it to the engine configurations tested and to aircraft altitude.

Most of the source assignment at the Barajas area could be attributed to clear differences between colder and warmer periods, without ruling out the influence of additional sources, particularly evident during the colder months. The mean B/T ratios were 0.36, 0.60 and 0.56 for October, November and December respectively. The differences for B/T ratio among these sampling periods are similar to those found by Jung et al, (2011) for summer (0.2) and winter (0.4) at the end of runaways of the Teterboro Airport, New Jersey (Fig. S7, ESM). These variations could be explained by the higher air temperature during the warm season. On the other hand, they could be due to multiple emissions from other sources. In both cases, the ratios would be lower, in line with the seasonal trends observed in urban areas (Yurdakul et al. 2018).

The mean X/E ratios were 2.09, 2.47 and 4.59 for October, November and December respectively. As mentioned above, the X/E ratio is employed in environmental studies to provide information on the intensity of the photochemical reactions i.e. as an indicator of the photochemical age of the air mass. A value of about 3.6 has been reported as the typical emission ratio for these compounds (Nelson and Quigley 1983), while in urban atmospheres it have been reported ratios close to 3 (Yurdakul et al. 2018). High values of these ratios (those closer to the expected emission ratio) typically indicate fresh local emissions, while low values suggest that the site is being influenced by emissions originated some distance away, the ageing of the air mass and effects of photochemical reactions. Ratios lower than 3 indicate the transport of VOCs from distant sources.

### Relationship between BTEX and PM

**Fig. 6:**
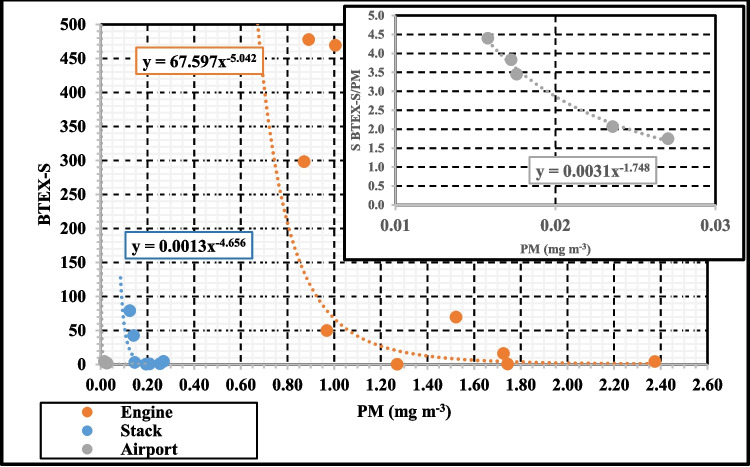
BTEX-sensitiveness (BTEX-S) vs PM concentration at three locations: engine plane, stack and airport

The BTEX-S concept was applied to the set of data at the three monitoring points: engine level, stack and airport environment, to assess the extent to which BTEX could be related to or influenced by PM. Figure 6 shows the coefficients calculated according to Eq. 2.

Comparing the three monitoring scenarios, it can be seen that the highest sensitivity of BTEX to PM corresponds to the airport. That is, in the engine plane and in the stack the slope of the BTEX-S vs PM curve is not as steep as in the case of the airport (Fig. S8, ESM).

In the airport environment, BTEX-S values are lower than those for emissions. The increase in BTEX concentration with respect to its background that could be related to an increase in PM concentration also with respect to its background, are lower than the one that would be required in the other scenarios. However, for the lowest concentrations measured in the stack, the slope of the sensitivity curve was close to that of the airport, which could be indicative of a transformation from BTEX, already in the plume, as previously observed.

The relationship between BTEX and PM in the engine primary emission is only appreciable for the lower PM concentrations, and only highly significant changes of the BTEX concentration could be related to small changes in PM.

At each of the sampling points, the higher values of the BTEX-S sensitivity coefficients were obtained for the lower PM concentrations, indicating that a greater change in the BTEX concentration is required to make a unit change of PM. In spite of the few available data, the results show that BTEXs are more sensitive to PM in the lower particulates ranges. This could indicate the most suitable and least polluting operating configurations, although at airports the PM concentration could be more easily affected by other factors or sources than by changes in BTEX concentrations during episodes of low PM concentrations.

Both measurement periods show that BTEX are more sensitive to PM in the lower particle size ranges. However, PM concentrations in the cold period (November–December) were more sensitive to the change of BTEX concentration than those in the warmer season (October), which could be related to the formation of new particles (Fig. S9, ESM).

From these results, we can evaluate how much PM concentrations can be affected by the change of VOC concentrations at the site of interest, contributing to establish policies for pollution controlling. By quantifying the extent to which VOC changes affect PM levels, policymakers can develop targeted strategies to mitigate air pollution and improve air quality, particularly in environments like airports where both BTEX and PM are prevalent. This could involve regulating VOC emissions from specific sources or implementing measures to reduce overall emissions during periods when PM concentrations are particularly sensitive to VOC changes.

## Conclusions

The findings of this study highlight the important role of BTEX emissions from aircraft engines in contributing to airport pollution, especially under specific operating conditions. The ground idle operation configuration that simulates airport taxiing manoeuvres (before take-off and after landing) has been identified as a critical contributor to BTEX emissions at airports. This configuration, being the longest phase within the landing and take-off (LTO) cycle, generates higher emissions compared to the take-off phase, despite the high fuel consumption associated with the latter. This highlights the importance of considering operational practices when assessing and managing air quality around airports.

Moreover, the analysis reveals that the prevalence of BTEX compounds in primary emissions remains consistent across various engine operations, with benzene consistently emitted at higher levels than toluene, followed by xylenes and ethylbenzene. While the emission of the former tend to increase with engine acceleration, the emissions of toluene, xylenes, and ethylbenzene diminish comparatively. This pattern indicates that although benzene is predominant in the initial emissions, its relative concentration can shift during subsequent flight phases, particularly in climbing and approach operations.

The mixing of the primary emission with ambient air in the plume not only causes the concentration of BTEX to decrease but also changes its prevalence at take-off, which leads us to think about possible sufficiently rapid physical–chemical reactions, when the temperature of the engines is high. This new order of prevalence is quite similar to that observed in ambient air near landing and take-off runways. In this case, benzene is no longer the main compound emitted, being toluene and xylenes the ones with the highest concentrations.

The variations of diagnostic ratios in the different study scenarios would confirm the transformation of BTEX compounds from their origin in the engine plane to the airport environment. Seasonal variations also play a role; lower benzene-to-toluene (B/T) ratios are typically noted during warmer months, which may be attributed to increased toluene evaporation at higher temperatures, even when engine temperatures have not stabilized. This seasonal dynamic highlights the influence of environmental conditions on emissions and their subsequent atmospheric behaviour.

Additionally, the sensitivity parameter against particle concentration indicates that a greater change in BTEX concentrations over its background value are required to induce a unit change in PM concentrations over theirs across all monitored scenarios. The data suggest that smaller particulate matter (PM) sizes exhibit greater sensitivity to fluctuations in BTEX levels, particularly during colder months. This could be indicative of new particle formation processes (such as Secondary Organic Aerosols—SOAs) occurring in the presence of BTEX emissions, emphasizing the complexity of atmospheric interactions at play.

Since the ground idle (GI) configuration generates the highest levels of BTEX emissions, it is recommended to implement operational practices that minimize taxiing time and optimize ground manoeuvres. This could include using tugs to tow aircraft instead of allowing engines to run on the ground for extended periods. Also, the use of alternative fuels in the near future is expected to reduce emissions of these compounds.

Given the critical findings of this study, further research is warranted in areas surrounding airports, particularly near terminals where travellers and airport personnel congregate. Such investigations would provide deeper insights into the evolution and presence of BTEX compounds and their relationship with other local pollution sources. By enhancing our understanding of these dynamics, effective strategies can be developed to mitigate pollution and protect public health in airport environments. Additionally, these findings can inform regulatory policies aimed at reducing emissions and improving air quality, particularly in vulnerable areas around airports. It would be needed to conduct additional studies to better understand the chemical transformations that occur between BTEX emissions and air quality in the airport environment. This could include investigating the role of temperature in the evolution of these emissions and the photochemical reactions.

## Supplementary Information

Below is the link to the electronic supplementary material.

ESM 1 (DOCX 3.85 MB)

## Data Availability

All data supporting the fndings of this study are available within the paper and its Supplementary information.
