# Supplementary material for: Transformation of BTEX compounds emitted by aircraft engines at ground level
Source: Environ Sci Pollut Res Int. 2025 Dec 2;32(51):29177–91. doi: 10.1007/s11356-025-37247-w (PMC12717120; doi:10.1007/s11356-025-37247-w)
Supplement: Supplementary file 1 — (DOCX 3.85 MB) [file 11356_2025_37247_MOESM1_ESM.docx]

Table 1S. Meteorological parameters: Wind speed, Temperature and Humidity, during Barajas campaigns.

| **Date and hour (sampling start and end) DD/MM/YY(h:min)** | **Mean wind speed**  **(East, m s^-1^)** | **Máx. wind speed**  **(East, m s^-1^)** | **SD wind speed (East, m s-1)** | **Mean wind speed**  **(West, m s-1)** | **Máx. wind speed**  **(West, m s^-1^)** | **S D wind speed**  **(West, m s-1)** | **Mean Temperature (ºC)** | **Máx./Min.T (ºC)** | **sd T (%)** | **Mean Humidity (%)** | **Máx. /Mín. Humidity (%)** | **sd Humidity(%)** |
| --- | --- | --- | --- | --- | --- | --- | --- | --- | --- | --- | --- | --- |
| **October campaign** | | | | | | | | | | | | |
| 08/10/2021 12:05 | 0.2 | 2.7 | 0.4 | 0.3 | 2.9 | 0.5 | 16.7 | 27.3/7.3 | 5.4 | 57.3 | 90.0/24.0 | 16.9 |
| 13/10/2021 11:58 | 0.8 | 4.6 | 1.2 | 0.1 | 1.29 | 0.2 | 15.1 | 26.0/6.2 | 6.8 | 44.3 | 72.0/14.0 | 19.4 |
| 13/10/2021 12:00 | 0.5 | 3.9 | 0.9 | 0.2 | 2.9 | 0.4 | 15.1 | 26.0/6.2 | 6.1 | 52.2 | 84.0/14.0 | 20.1 |
| 14/10/2021 12:09 | 0.1 | 1.3 | 0.2 | 0.3 | 2.9 | 0.5 | 15.3 | 24.3/8.0 | 5.3 | 59.6 | 84.0/29.0 | 17.4 |
| 15/10/2021 11:48 | 0.2 | 2.0 | 0.4 | 0.3 | 3.55 | 0.6 | 16.2 | 24.8/7.8 | 4.5 | 66.7 | 98.0/24.8 | 19.1 |
| 15/10/2021 12:02 | 0.2 | 2.0 | 0.4 | 0.3 | 3.55 | 0.6 | 16.2 | 24.8/7.80 | 4.5 | 66.8 | 98.0/31.0 | 19.1 |
| 18/10/2021 11:41 | 0.2 | 2.4 | 0.4 | 0.2 | 1.58 | 0.4 | 17.3 | 25.4/10.1 | 4.6 | 68.6 | 92.0/41.0 | 16.7 |
| 18/10/2021 12:02 | 0.2 | 2.4 | 0.4 | 0.2 | 1.58 | 0.4 | 17.3 | 25.4/10.1 | 4.6 | 68.7 | 92.0/41.0 | 16.7 |
| 18/10/2021 12:08 | 0.2 | 2.4 | 0.4 | 0.3 | 1.58 | 0.4 | 17.4 | 24.5/11.4 | 4.2 | 69.2 | 92.0/42.0 | 16.8 |
| 20/10/2021 11:30 | 0.8 | 6.7 | 1.6 | 1.2 | 8.11 | 1.9 | 17.2 | 24.9/10.6 | 4.1 | 58.1 | 91.0/36.0 | 13.8 |
| 20/10/2021 12:00 | 0.9 | 6.7 | 1.6 | 1.3 | 8.11 | 1.9 | 17.2 | 24.9/10.6 | 4.1 | 58.1 | 91.0/36.0 | 13.8 |
| 20/10/2021 12:27 | 0.2 | 1.7 | 0.3 | 2.2 | 8.11 | 2.3 | 18.3 | 24.9/10.6 | 4.3 | 63.6 | 91.0/43.0 | 14.9 |
| 22/10/2021 11:19 | 0.9 | 8.5 | 1.8 | 0.2 | 2.67 | 0.5 | 11.7 | 21.1/2.2 | 5.8 | 57.0 | 88.0/27.0 | 18.8 |
| 22/10/2021 11:47 | 0.9 | 8.5 | 1.8 | 0.2 | 2.67 | 0.5 | 11.7 | 21.1/2.2 | 5.8 | 57.0 | 88.0/27.0 | 18.8 |
| 22/10/2021 11:55 | 1.2 | 8.5 | 2.1 | 0.2 | 2.06 | 0.4 | 11.7 | 20.3/2.2 | 5.6 | 57.8 | 88.0/30.0 | 18.1 |
| 25/10/2021 11:30 | 0.2 | 1.9 | 0.4 | 0.3 | 2.53 | 0.4 | 12.6 | 21.5/4.7 | 5.3 | 57.3 | 84.0/28.0 | 16.1 |
| 25/10/2021 12:02 | 0.2 | 1.9 | 0.4 | 0.3 | 2.53 | 0.4 | 12.5 | 21.5/4.7 | 5.3 | 57.6 | 84.0/28.0 | 16.0 |
| 26/10/2021 12:15 | 0.2 | 1.5 | 0.3 | 0.3 | 2.42 | 0.4 | 12.4 | 21.5/4.7 | 5.5 | 60.8 | 84.0/35.0 | 15.3 |
| 27/10/2021 12:00 | 0.3 | 2.0 | 0.5 | 0.7 | 4.83 | 1.1 | 14.0 | 21.4/5.7 | 4.7 | 57.6 | 84.0/28.0 | 16.0 |
| 28/10/2021 12:31 | 0.3 | 2.0 | 0.5 | 1.3 | 4.83 | 1.3 | 11.7 | 21.0/12.6 | 2.5 | 51.6 | 82.0/29.0 | 11.8 |

Table 2S. Ambient measurements of Wind speed, Temperature and Humidity during Barajas campaign.

| **Date and hour (sampling start and end) DD/MM/YY(h:min)** | **Mean wind speed**  **(East, m s^-1^)** | **Máx. wind speed**  **(East, m s^-1^)** | **SD wind speed (East, m s-1)** | **Mean wind speed**  **(West, m s-1)** | **Máx. wind speed**  **(West, m s^-1^)** | **S D wind speed**  **(West, m s-1)** | **Mean Temperature (ºC)** | **Máx./Min.T (ºC)** | **sd T (%)** | **Mean Humidity (%)** | **Máx. /Mín. Humidity (%)** | **sd Humidity**  **(%)** |
| --- | --- | --- | --- | --- | --- | --- | --- | --- | --- | --- | --- | --- |
| **November-December Campaign** | | | | | | | | | | | | |
| 19/11/2021 11:41 | 0.8 | 4.0 | 0.9 | 0.2 | 3.1 | 0.5 | 10.2 | 16.7/6.0 | 2.5 | 80.9 | 99.0/47.0 | 13.3 |
| 24/11/2021 10:57 | 0.5 | 3.2 | 0.7 | 0.5 | 4.8 | 1.0 | 5.1 | 11.2/1.0 | 2.7 | 78.0 | 98.0/47.0 | 13.9 |
| 24/11/2021 12:00 | 0.5 | 3.2 | 0.7 | 0.6 | 4.8 | 1.0 | 5.1 | 11.2/1.0 | 2.8 | 77.6 | 98.0/47.0 | 14.4 |
| 26/11/2021 12:03 | 0.4 | 5.1 | 0.9 | 0.5 | 10.6 | 2.5 | 4.5 | 10.8/-2.7 | 3.3 | 66.5 | 89.0/39.0 | 12.9 |
| 29/11/2021 11:30 | 0.2 | 3.4 | 0.4 | 0.8 | 6.7 | 1.5 | 5.6 | 15.0/-1.8 | 5.3 | 77.8 | 99.0/48.0 | 17.9 |
| 29/11/2021 12:00 | 0.2 | 3.4 | 0.4 | 0.8 | 6.7 | 1.5 | 5.5 | 15.0/-1.8 | 5.2 | 78.1 | 99.0/48.0 | 17.8 |
| 03/12/2021 11:34 | 0.2 | 2.2 | 0.5 | 2.8 | 10.3 | 3.1 | 7.2 | 13.9/-2.2 | 4.1 | 67.5 | 97.0/40.0 | 15.5 |
| 03/12/2021 11:50 | 0.2 | 3.6 | 0.5 | 3.7 | 13.7 | 3.3 | 7.6 | 14.6/-2.2 | 3.6 | 69.9 | 99.0/40.0 | 16.2 |
| 03/12/2021 12:00 | 0.3 | 1.8 | 0.4 | 0.3 | 2.5 | 0.5 | 4.6 | 13.1/-2.2 | 5.1 | 76.1 | 97.0/40.0 | 18.9 |
| 09/12/2021 11:30 | 0.0 | 1.5 | 0.2 | 6.3 | 13.9 | 3.1 | 11.3 | 15.3/7.4 | 1.7 | 65.8 | 83.0/56.0 | 5.2 |
| 09/12/2021 11:45 | 0.0 | 1.5 | 0.2 | 6.3 | 13.9 | 3.1 | 11.4 | 15.8/7.4 | 1.8 | 65.6 | 83.0/54.0 | 5.4 |
| 10/12/2021 11:41 | 0.3 | 3.6 | 0.5 | 1.4 | 10.8 | 2.8 | 8.5 | 18.2/-0.9 | 5.1 | 74.5 | 98.0/45.0 | 17.5 |
| 10/12/2021 12:04 | 0.3 | 3.6 | 0.5 | 1.4 | 10.8 | 2.8 | 8.4 | 18.2/-0.9 | 5.1 | 74.7 | 98.0/45.0 | 17.6 |
| 11/12/2021 12:10 | 0.2 | 2 | 0.4 | 0.2 | 2.2 | 0.4 | 6.9 | 18.2/-0.9 | 5.1 | 82.8 | 98.0/45.0 | 15.2 |
| 13/12/2021 11:55 | 0.1 | 1.7 | 0.3 | 0.3 | 1.9 | 0.4 | 5.6 | 17.8/-1.5 | 5.5 | 77.5 | 95.0/41.0 | 14.7 |
| 13/12/2021 12:37 | 0.2 | 1.7 | 0.3 | 0.2 | 1.9 | 0.4 | 5.9 | 17.8/-1.5 | 5.5 | 76.8 | 95.0/41.0 | 14.9 |

Table 3S. BTEX on plane engine exhaust emissions for the test configurations (INTA).

| Date | Aircraft Engine Configuration | CO_2_ (ppm) * | BTEX (ng L^-1^) | | | | | |
| --- | --- | --- | --- | --- | --- | --- | --- | --- |
|  |  |  | Benzene | Toluene | Ethylbenzene | m+p-xylene | o-xylene | ΣBTEX |
| 04/06/2021 | Ground Idle | 520 | 113 | 30 | 9.4 | 15 | 9.7 | 177 |
|  |  | 632 | 221 | 38 | 3.3 | 9.2 | 4.2 | 275 |
| 06/06/2021 | Take off | 2288 | <0.05 | 1.0 | <0.05 | <0.05 | <0.05 | 1.0 |
| 07/06/2021 | Ground Idle to Take off | 1116 | 19 | 3.9 | 0.54 | 1.3 | 0.57 | 25 |
|  |  | 1223 | 133 | 23 | 2.5 | 6.2 | 2.8 | 168 |
|  |  | 1619 | 7.9 | 6.4 | 0.43 | 1.2 | 0.50 | 16 |
|  | Take-off | 1914 | 84 | 17 | 2.0 | 7.5 | 2.2 | 112 |
|  | Take off  to Climb-out | 1071 | 120 | 12 | 0.64 | 1.8 | 0.78 | 135 |
| 11/06/2021 | Ground Idle | 464 | 98 | 56 | 16 | 28 | 15 | 213 |
|  | Ground Idle to  Take off | 982 | 2.6 | 1.0 | <0.05 | <0.05 | <0.05 | 3.6 |
|  | Ground Idle | 1371 | 807 | 233 | 40 | 96 | 45 | 1221 |
|  |  | 1016 | 476 | 199 | 95 | 74 | 27 | 871 |
|  | Ground Idle to  Take off | 1391 | 7.7 | 5.4 | 0.36 | 2.0 | 1.1 | 17 |

**Measurements of CO_2_ in stack.*

**Ground idle**

**Ground idle to take-off**

**Take-off**

**Take-off to climb-out**

Benzene……………...dark colour

Toluene

Ethylbenzene

m+p xylene

o-xylene………….. light colour

Figure 1S. BTEX emission vs Engine operation in the different test configuration (INTA, 2021).

Table 4S. BTEX emissions at the engine exit plane and test conditions in summer and winter (CRIA).

| Date | Aircraft Engine Configuration | CO_2_ (ppm) | BTEX (ng L^-1^) | | | | | |
| --- | --- | --- | --- | --- | --- | --- | --- | --- |
|  |  |  | Benzene | Toluene | Ethylbenzene | m+p-xylene | o-xylene | ΣBTEX |
| 26/07/2021 | JET A1 | 548 | 6.4 | 2.6 | 0.70 | 3.1 | 1.4 | 14 |
|  | Flight Idle | 548 | 4.8 | 2.4 | 0.63 | 3.0 | 1.4 | 12 |
|  |  | 548 | 5.3 | 2.2 | 0.60 | 2.8 | 1.3 | 12 |
|  | JET A1 | 793 | 41 | 7.8 | 0.74 | 2.5 | 1.1 | 53 |
|  | Ground Idle | 793 | 39 | 7.6 | 0.74 | 2.6 | 1.2 | 51 |
|  | (65% N3) | 793 | 39 | 8.9 | 0.87 | 2.9 | 1.3 | 53 |
| 22/01/2022 | JET A1 | 809 | na | 3.2 | 0.57 | 1.5 | 0.67 | 6 |
|  | Flight Idle | 807 | 25 | 5.4 | 0.50 | 0.59 | 0.50 | 32 |
|  | JET A1  30 %N1 | 988 | 1.7 | 0.69 | 0.50 | 0.50 | 0.50 | 3.9 |
| 25/01/2022 | SAF | 563 | 7.9 | 2.2 | 0.30 | 1.2 | 0.50 | 12 |
|  | Flight Idle |  |  |  |  |  |  |  |
|  | SAF  30%N1 | 822 | 1.3 | 0.46 | <0.05 | 0.41 | 0.16 | 2.3 |
|  |  | 822 | 1.4 | 0.48 | <0.05 | 0.42 | 0.15 | 2.5 |
| 26/01/2022 | SAF | 721 | 86 | 24 | 4.0 | 12 | 5.2 | 131 |
|  | Ground Idle | 721 | 136 | 44 | 8.0 | 23 | 10 | 221 |
|  | (62% N3) | 721 | 147 | 44 | 7.5 | 22 | 9.5 | 230 |

|  | |
| --- | --- |
| Jet A1 | Jet A1+SAF  Jet A1 |
| **SUMMER** | **WINTER** |

*Figure 2S. BTEX concentrations, from one of aircraft engines, of an AIRBUS 340, in summer and winter at CRIA.*

Table 5S. BTEX in the emissions at stack for test configurations (INTA).

| Date | Aircraft Engine Configuration | CO_2_ (ppm)** | BTEX (ng L^-1^) | | | | | |
| --- | --- | --- | --- | --- | --- | --- | --- | --- |
|  |  |  | Benzene | Toluene | Ethylbenzene | m+p-xylene | o-xylene | ΣBTEX |
| 07/06/2021 | Ground Idle | 1068 | 0.74 | 0.54 | 0.21 | 1.9 | 0.97 | 4.4 |
| 10/06/2021 |  |  | 0.45 | 1.3 | <0.05 | 0.52 | 0.24 | 2.5 |
|  |  |  | 1.3 | 2.0 | <0.05 | 0.59 | 0.27 | 4.2 |
| 14/06/2021 | Take off (*) |  | 2.0 | 6.2 | 1.3 | 6.2 | 3.1 | 19 |
|  | Ground Idle (*) | 1327 | 3.6 | 4.2 | 0.66 | 3.9 | 2.0 | 14 |
|  |  | 1337 | 2.6 | 4.1 | 0.68 | 3.6 | 1.6 | 12 |
|  | Ground Idle to | 1522 | 2.6 | 3.7 | 0.58 | 3.0 | 0.89 | 11 |
|  | Take off (*) | 1892 | 0.69 | 0.67 | <0.05 | <0.05 | 0.26 | 1.6 |
|  | Take off (*) | 2053 | 0.16 | 0.47 | <0.05 | <0.05 | 0.26 | 0.89 |
| 16/06/2021 | Ground Idle (*) | 1113 | 3.0 | 0.45 | <0.05 | <0.05 | 0.26 | 3.7 |
|  | Ground Idle to | 1338 | 1.5 | 0.12 | <0.05 | <0.05 | 0.2 | 1.8 |
|  | Take off (*) |  |  |  |  |  |  |  |
|  | Take off (*) | 1948 | 0.14 | <0.05 | <0.05 | <0.05 | 0.13 | 0.27 |
| 18/06/2021 | Ground Idle (*) | 1071 | 5.0 | 1.3 | 0.21 | 1.5 | 0.92 | 8.9 |
|  |  | 1155 |  |  |  |  |  |  |
|  | Ground Idle to | 1444 | 1.1 | 0.65 | <0.05 | 1.0 | 0.76 | 3.5 |
|  | Take off (*) |  |  |  |  |  |  |  |
|  | Take off (*) | 1075 | 0.22 | 0.28 | <0.05 | 0.70 | 0.62 | 1.8 |
|  | Ground Idle (*) | 1395 | 3.0 | 0.54 | <0.05 | 0.64 | 0.61 | 4.7 |
|  | Ground Idle to |  | 0.50 | <0.05 | <0.05 | 1.0 | 0.58 | 2.1 |
|  | Take off (*) |  |  |  |  |  |  |  |
|  | Take off (*) | 1068 | 0.31 | <0.05 | <0.05 | 0.24 | 0.42 | 0.97 |

*With breather

***Measurements of CO_2_ in stack.*

|  |  |
| --- | --- |

*Figure 3S. PM and BTEX mean concentration from emission Engine plane and Stack at INTA*

**Ground idle**

**Ground idle to take-off**

**Take-off**

Benzene……………...dark

Toluene

Ethylbenzene

m+p xylene

o-xylene………….. clear

*Figure 4S. BTEX emission vs Engine operation from stack in the different test configuration (INTA)*

*Figure 5S. BTEX at Barajas Airport: a) October and b) November and December*

| Table 6S. BTEX emissions around runways at Barajas airport, (October) |
| --- |
| \| **OCTOBER** \| \| \| \| \| \| \| \| \| --- \| --- \| --- \| --- \| --- \| --- \| --- \| --- \| \| **DATE** \| **Time(h)** \| **BTEX (ng L^-1^)** \| \| \| \| \| \| \| **Benzene** \| **Toluene** \| **Ethylbenzene** \| **m+p-xylene** \| **o-xylene** \| **ΣBTEX** \| \| 08/10/2021 \| 12:00 \| 2.0 \| 8.4 \| 0.59 \| 0.87 \| 0.38 \| 12 \| \| 20:00 \| 0.61 \| 3.1 \| 0.25 \| 0.4 \| 0.18 \| 4.6 \| \| 09/10/2021 \| 4:00 \| 0.78 \| 2.0 \| 0.15 \| 0.21 \| 0.12 \| 3.3 \| \| 11/10/2021 \| 12:00 \| 0.77 \| 3.9 \| 0.24 \| 0.31 \| 0.13 \| 5.3 \| \| 20:00 \| 0.80 \| 1.8 \| 0.13 \| 0.22 \| 0.11 \| 3.1 \| \| 12/10/2021 \| 4:00 \| 1.4 \| 1.6 \| 0.12 \| 0.16 \| 0.08 \| 3.3 \| \| 13/10/2021 \| 12:00 \| 0.49 \| 1.7 \| 0.18 \| 0.35 \| 0.13 \| 2.9 \| \| 20:00 \| 0.62 \| 1.5 \| 0.18 \| 0.46 \| 0.18 \| 2.9 \| \| 14/10/2021 \| 4:00 \| 0.76 \| 1.3 \| 0.14 \| 0.31 \| 0.10 \| 2.6 \| \| 12:00 \| 0.22 \| 2.4 \| 0.30 \| 0.56 \| 0.22 \| 3.7 \| \| 20:00 \| 0.22 \| 3.7 \| 0.38 \| 0.76 \| 0.32 \| 5.3 \| \| 15/10/2021 \| 4:00 \| 0.41 \| 2.1 \| 0.26 \| 0.41 \| 0.18 \| 3.3 \| \| 12:00 \| 0.44 \| 6.6 \| 0.8 \| 1.28 \| 0.56 \| 9.7 \| \| 20:00 \| 0.24 \| 2.6 \| 0.4 \| 0.93 \| 0.41 \| 4.5 \| \| 4:00 \| 0.41 \| 2.3 \| 0.37 \| 0.65 \| 0.3 \| 4.1 \| \| 18/10/2021 \| 12:00 \| 0.48 \| 7.2 \| 0.71 \| 1.3 \| 0.54 \| 10.2 \| \| 20:00 \| 0.64 \| 1.8 \| 0.38 \| 0.91 \| 0.40 \| 4.2 \| \| 19/10/2021 \| 4:00 \| 0.71 \| 1.9 \| 0.37 \| 0.77 \| 0.33 \| 4.1 \| \| 12:00 \| 0.44 \| 3.8 \| 0.59 \| 1.24 \| 0.53 \| 6.6 \| \| 20:00 \| 0.41 \| 2.56 \| 0.42 \| 1.07 \| 0.47 \| 4.9 \| \| 20/10/2021 \| 4:00 \| 0.96 \| 1.9 \| 0.37 \| 0.8 \| 0.33 \| 4.3 \| \| 12:00 \| 0.67 \| 5.8 \| 1.0 \| 1.7 \| 0.77 \| 10 \| \| 20:00 \| 0.50 \| 2.8 \| 0.50 \| 1.4 \| 0.59 \| 5.8 \| \| 21/10/2021 \| 4:00 \| 0.56 \| 2.5 \| 0.34 \| 0.79 \| 0.33 \| 4.5 \| \| 12:00 \| 0.08 \| 2.2 \| 0.37 \| 0.88 \| 0.42 \| 4.0 \| \| 12:00 \| 0.7 \| 5.1 \| 0.94 \| 2.3 \| 0.82 \| 9.9 \| \| 20:00 \| 0.41 \| 2.7 \| 0.48 \| 1.1 \| 0.49 \| 5.3 \| \| 22/10/2021 \| 4:00 \| 0.42 \| 0.88 \| 0.15 \| 0.37 \| 0.16 \| 2.0 \| \| 12:00 \| 0.29 \| 0.07 \| <0.05 \| <0.05 \| <0.05 \| 0.36 \| \| 12:00 \| 0.42 \| 0.97 \| 0.15 \| 0.38 \| 0.17 \| 2.1 \| \| 20:00 \| 0.25 \| 0.79 \| 0.12 \| 0.37 \| 0.17 \| 1.7 \| \| 23/10/2021 \| 4:00 \| 0.43 \| 0.7 \| 0.09 \| 0.18 \| 0.09 \| 1.5 \| \| 25/10/2021 \| 12:00 \| 1.0 \| 4.2 \| 0.65 \| 1.1 \| 0.50 \| 7.5 \| \| 20:00 \| 0.88 \| 3.9 \| 0.54 \| 1.3 \| 0.61 \| 7.3 \| \| 26/10/2021 \| 4:00 \| 0.93 \| 1.9 \| 0.3 \| 0.6 \| 0.28 \| 4.0 \| \| 12:00 \| 0.79 \| 2.0 \| 0.44 \| 0.99 \| 0.42 \| 4.7 \| \| 20:00 \| 0.77 \| 3.2 \| 0.37 \| 0.95 \| 0.43 \| 5.8 \| \| 27/10/2021 \| 4:00 \| 0.61 \| 2.1 \| 0.32 \| 0.66 \| 0.28 \| 3.9 \| \| 12:00 \| 0.75 \| 4.0 \| 0.72 \| 1.5 \| 0.65 \| 7.6 \| \| 20:00 \| 0.59 \| 2.4 \| 0.63 \| 1.5 \| 0.68 \| 5.8 \| \| 28/10/2021 \| 4:00 \| 0.83 \| 2.6 \| 0.40 \| 0.78 \| 0.34 \| 4.9 \| \| 12:00 \| 0.98 \| 4.0 \| 0.71 \| 1.6 \| 0.69 \| 8.0 \| \| 20:00 \| 0.78 \| 2.4 \| 0.55 \| 1.4 \| 0.59 \| 5.7 \| \| 29/10/2021 \| 12:00 \| 0.95 \| 4.1 \| 0.63 \| 1.5 \| 0.66 \| 7.9 \| \| 12:00 \| 0.74 \| 3.2 \| 0.69 \| 1.8 \| 0.83 \| 7.27 \| |

| Table 7S. BTEX emissions around runways at Barajas airport, (November -December) |
| --- |
| \| **November-December** \| \| \| \| \| \| \| \| \| --- \| --- \| --- \| --- \| --- \| --- \| --- \| --- \| \| **DATE** \| **Time (h)** \| **BTEX (ng L^-1^)** \| \| \| \| \| \| \| **Benzene** \| **Toluene** \| **Ethylbenzene** \| **m+p-xylene** \| **o-xylene** \| **ΣBTEX** \| \| 19/11/2021 \| 12:00 \| 0.52 \| 1.6 \| 0.29 \| 0.60 \| 0.27 \| 3.3 \| \| 20:00 \| 0.61 \| 1.4 \| 0.35 \| 0.87 \| 0.33 \| 3.6 \| \| 4:00 \| 0.72 \| 0.97 \| 0.18 \| 0.47 \| 0.19 \| 2.5 \| \| 22/11/2021 \| 12:00 \| 0.67 \| 0.96 \| 0.2 \| 0.48 \| 0.21 \| 2.5 \| \| 24/11/2021 \| 12:00 \| 1.38 \| 3.8 \| 0.75 \| 1.83 \| 0.82 \| 8.6 \| \| 20:00 \| 0.72 \| 2.61 \| 0.44 \| 1.12 \| 0.49 \| 5.4 \| \| 25/11/2021 \| 4:00 \| 1.0 \| 3.1 \| 0.86 \| 2.2 \| 1.000 \| 8.1 \| \| 12:00 \| 0.87 \| 4.5 \| 0.82 \| 2.3 \| 1.0 \| 9.4 \| \| 20:00 \| 0.37 \| 0.54 \| 0.11 \| 0.29 \| 0.13 \| 1.4 \| \| 26/11/2021 \| 4:00 \| 0.42 \| 0.72 \| 0.13 \| 0.32 \| 0.15 \| 1.7 \| \| 12:00 \| 0.57 \| 1.21 \| 0.27 \| 0.6 \| 0.31 \| 3.0 \| \| 20:00 \| 0.96 \| 2.65 \| 0.71 \| 1.6 \| 0.77 \| 6.7 \| \| 27/11/2021 \| 4:00 \| 0.69 \| 1.7 \| 0.35 \| 0.92 \| 0.45 \| 4.1 \| \| 12:10 \| 0.38 \| 0.38 \| 0.09 \| 0.22 \| 0.14 \| 1.2 \| \| 20:10 \| 0.63 \| 0.46 \| 0.08 \| 0.15 \| 0.09 \| 1.4 \| \| 28/11/2021 \| 4:10 \| 0.57 \| 0.86 \| 0.17 \| 0.35 \| 0.19 \| 2.1 \| \| 29/11/2021 \| 12:00 \| 0.48 \| 0.40 \| 0.10 \| 0.29 \| 0.13 \| 1.4 \| \| 20:00 \| 0.50 \| 0.32 \| 0.11 \| 0.30 \| 0.13 \| 1.4 \| \| 30/11/2021 \| 4:00 \| 0.48 \| 0.91 \| 0.16 \| 0.40 \| 0.18 \| 2.1 \| \| 12:00 \| 0.4 \| 2.1 \| 0.48 \| 1.3 \| 0.60 \| 4.9 \| \| 20:00 \| 0.89 \| 5.1 \| 0.88 \| 2.4 \| 1.07 \| 10 \| \| 01/12/2021 \| 4:00 \| 0.5 \| 0.99 \| 0.19 \| 0.46 \| 0.21 \| 2.4 \| \| 03/12/2021 \| 12:00 \| 2.0 \| 6.6 \| 2.0 \| 3.9 \| 1.7 \| 16 \| \| 20:00 \| 1.2 \| 4.3 \| 0.75 \| 2.1 \| 0.92 \| 9.3 \| \| 04/12/2021 \| 4:00 \| 1.1 \| 2.3 \| 0.47 \| 1.3 \| 0.51 \| 5.6 \| \| 09/12/2021 \| 12:00 \| 0.33 \| 0.54 \| 0.12 \| 0.29 \| 0.13 \| 1.4 \| \| 20:00 \| 0.30 \| 0.26 \| 0.06 \| 0.16 \| 0.08 \| 0.86 \| \| 10/12/2021 \| 4:00 \| 0.53 \| 0.71 \| 0.12 \| 0.34 \| 0.16 \| 1.9 \| \| 12:36 \| 0.41 \| 0.86 \| 0.20 \| 0.56 \| 0.26 \| 2.3 \| \| 20:36 \| 0.18 \| 0.15 \| <0.05 \| 0.09 \| 0.05 \| 0.47 \| \| 11/12/2021 \| 4:36 \| 0.26 \| 0.72 \| 0.12 \| 0.32 \| 0.15 \| 1.6 \| \| 12:40 \| 0.44 \| 1.1 \| 0.31 \| 0.89 \| 0.42 \| 3.1 \| \| 20:49 \| 0.27 \| 0.56 \| 0.13 \| 0.36 \| 0.17 \| 1.5 \| \| 12/12/2021 \| 4:40 \| 0.30 \| 2.3 \| 0.20 \| 0.53 \| 0.22 \| 3.5 \| \| 13/12/2021 \| 13:02 \| 0.25 \| 1.1 \| 0.27 \| 0.71 \| 0.35 \| 2.7 \| \| 20:02 \| 0.07 \| <0.04 \| <0.05 \| <0.05 \| <0.05 \| 0.07 \| \| 14/12/2021 \| 4:02 \| <0.05 \| <0.04 \| <0.05 \| <0.05 \| <0.05 \| 0 \| \| 12:10 \| 0.30 \| 1.2 \| 0.27 \| 0.75 \| 0.30 \| 2.9 \| \| 20:10 \| 0.34 \| 1.4 \| 0.28 \| 0.77 \| 0.33 \| 3.1 \| \| 15/12/2021 \| 4:10 \| 0.32 \| 0.74 \| 0.13 \| 0.32 \| 0.15 \| 1.7 \| |


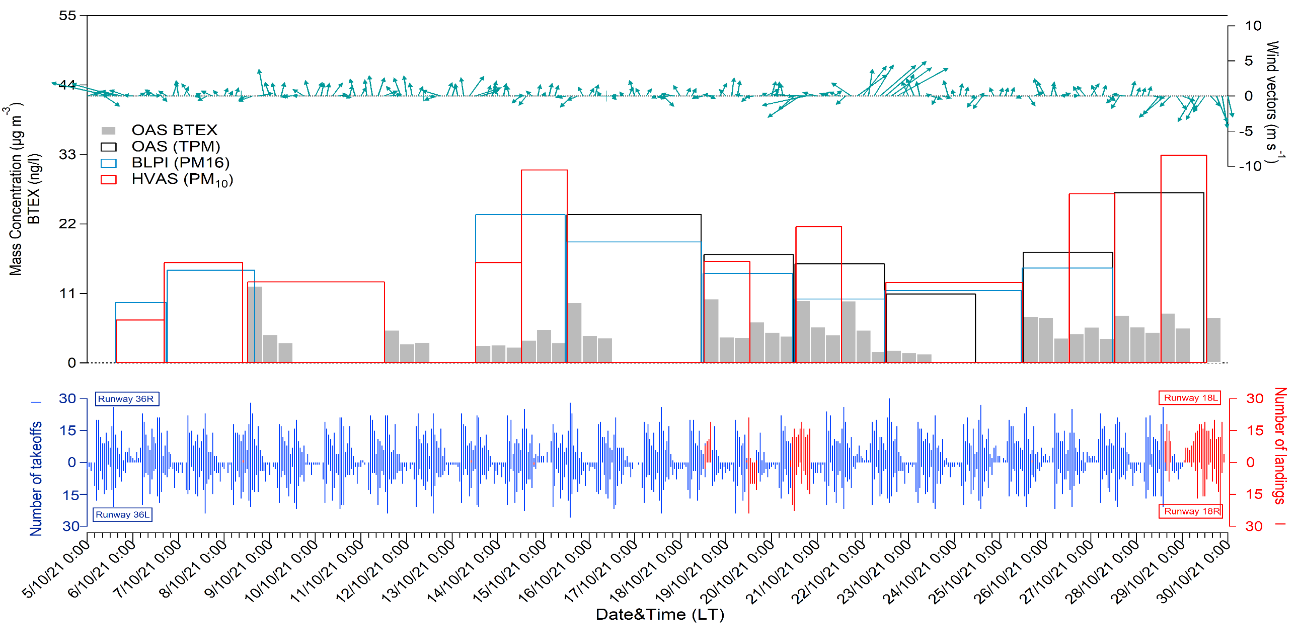


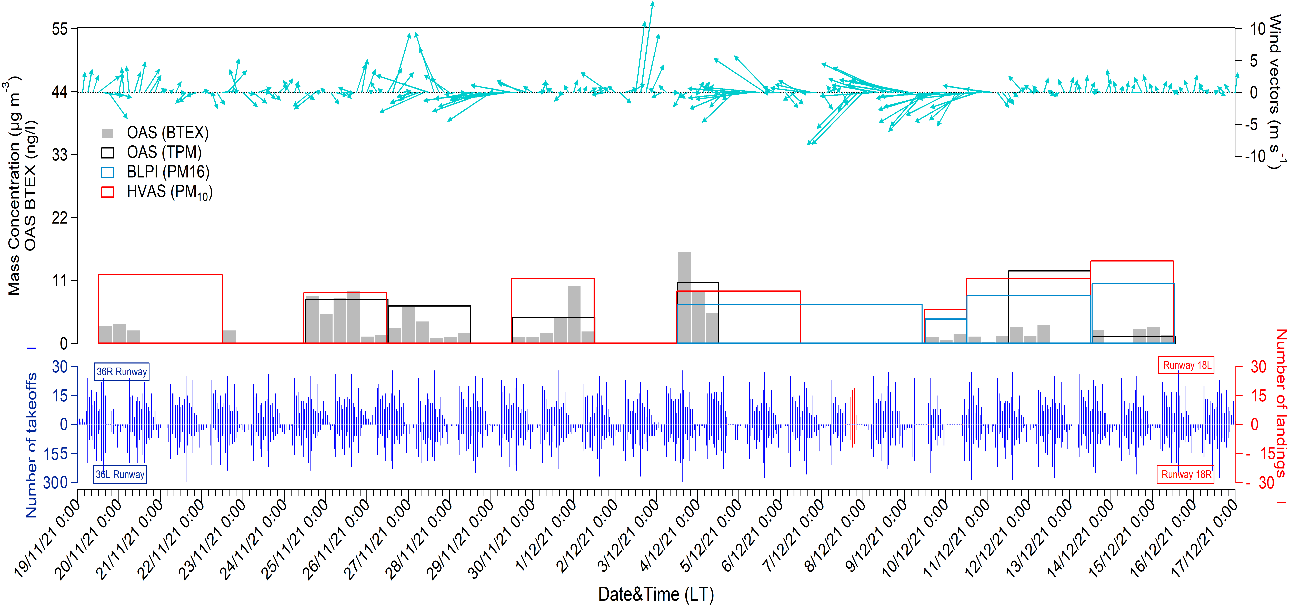


*Figure 6S. BTEX, PM, meteorological parameters and flights in October and Nov-Dec campaigns at Barajas airport.*

Table 8S. B/T and X/E ratios on plane engine exhaust emissions (INTA and CRIA).

| Engine (INTA) | | | | Engine (CRIA) | | | |
| --- | --- | --- | --- | --- | --- | --- | --- |
| Date | Aircraft Engine Configuration | B/T | X/E | Date | Aircraft Engine Configuration | B/T | X/E |
| 04/06 | Ground Idle | 3.82 | 1.63 | 26/07 | JET A1  Flight Idle | 2.46 | 4.43 |
|  |  | 5.88 | 2.83 |  |  | 2.05 | 4.81 |
| 06/06 | Take off | 0.06 | 0.50 |  |  | 2.43 | 4.67 |
| 07/06 | Ground Idle to Take off | 4.76 | 2.33 |  | JET A1  Ground Idle  (65% N3) | 5.33 | 3.42 |
|  |  | 5.78 | 2.49 |  |  | 5.09 | 3.54 |
|  |  | 1.23 | 2.67 |  |  | 4.39 | 3.36 |
|  | Take-off | 5.09 | 2.36 | 22/01 | JET A1  Flight Idle | 4.64 | 1.18 |
|  | Take off  to Climb-out | 10.07 | 2.81 |  | JET A1  30 %N1 | 2.46 | 1.00 |
|  |  |  |  | 25/01 | SAF  Flight Idle | 3.66 | 4.13 |
| 11/06 | Ground Idle | 1.75 | 1.79 |  | SAF  30%N1 | 2.76 | 5.13 |
|  | Ground Idle to  Take off | 2.63 | - |  |  | 3.00 | 4.67 |
|  | Ground Idle | 3.47 | 2.43 | 26/01 | SAF  Ground Idle  (62% N3) | 3.54 | 2.93 |
|  |  | 2.39 | 0.79 |  |  | 3.06 | 2.84 |
|  | Ground Idle to  Take off | 1.43 | 5.50 |  |  | 3.34 | 2.86 |

Table 9S. B/T and X/E ratios in stack emissions (INTA) and ambient air (Barajas airport).

| Stack (INTA) | | | | Barajas Airport | | | |
| --- | --- | --- | --- | --- | --- | --- | --- |
| Date | Aircraft Engine Configuration | B/T | X/E | Month | Statistic | B/T | X/E |
| 07/06 | Ground Idle (**) | 1.37 | 9.24 | October | Mean | 0.36 | 2.09 |
| 10/06 |  | 0.36 | 8.67 |  | Max. | 4.14 | 3.08 |
|  |  | 0.15 | 5.90 |  | Min. | 0.04 | 1.00 |
| 14/06 | Take off (*) | 0.32 | 4.63 | November | Mean | 0.60 | 2.47 |
|  | Ground Idle (*) | 0.85 | 5.95 |  | Max. | 1.56 | 2.90 |
|  |  | 0.63 | 5.24 |  | Min. | 0.17 | 1.88 |
|  | Ground Idle to take off (*) | 0.70 | 5.24 | December | Mean | 0.56 | 4.59 |
| 18/06 | Ground Idle (*) | 3.81 | 7.05 |  | Max. | 1.75 | 11.40 |
|  | Ground Idle to Take off (*) | 1.66 | 20.20 |  | Min. | 0.13 | 0.80 |
|  | Take off (*) | 0.79 | 14.00 |  |  |  |  |
|  | Ground Idle to take off (*) | 1.37 | 9.24 |  |  |  |  |
|  | Take off (*) | 0.36 | 8.67 |  |  |  |  |

* With breather

**Without breather

*Figure 7S. Benzene/toluene and m+p xylene/ethylbenzene ratios for Barajas airport.*

*Figure 8S. Sensibility curve Slope vs dp at Engine plane (INTA), Stack (INTA) and Airport.*

| **October** | **Nov-Dec** |
| --- | --- |

*Figure 9S. Sensitivity coefficients for PM different size ranges, in October and November-December.*
